# Clinico-pathological characteristics of obstructing colorectal cancer and its management outcomes at a tertiary referral center of Eastern Nepal

**DOI:** 10.1186/s12876-022-02380-0

**Published:** 2022-06-21

**Authors:** Abhijeet Kumar, Sajan Babu Dhungana, Rakesh Kumar Gupta, Suresh Prasad Sah, Bhawani khanal

**Affiliations:** 1 Department of Surgery, B. P. Koirala Institute of Health Sciences, Dharan, Nepal; 2 B. P. Koirala Institute of Health Sciences, Dharan, Nepal

**Keywords:** Obstructed colorectal cancer (OCRC), Partial obstruction, Complete obstruction, Anatomical shift, Developing country

## Abstract

**Background:**

The aim of this study is to explore the treatment strategies being followed for patients with obstructing colorectal cancer (OCRC) at our institute and to know the management outcomes.

**Methods:**

This study included 28 patients who were diagnosed with obstructing colorectal cancer (OCRC) either preoperatively or intraoperatively over a period of 5 years.

**Results:**

Most were in the younger age group with mean age of 49.78 ± 15.96 years with 1/4th of the patients being younger than 40. There was no difference in incidence of OCRC among genders. It was found to be common in rural areas of the eastern Nepal, 16(57%) patients from such areas. 21.4% patients had complete bowel obstruction at presentation. The investigating modalities used were abdominal X-ray, ultrasonoghraphy of abdomen/pelvis, abdominal CT-scan, colonoscopy, serum CEA, punch biopsy and Faecal occult blood test. The anatomical shift to the right was observed with 54% lesions in the proximal colon. Majority were in advanced stage (stage 3:53.6%, stage 4:32.1%) with histologically adenocarcinoma (100%) and a higher incidence of synchronous lesion (28.6%). Patients averaged 13.82 days in the hospital with post-operative mortality rate of 3.6%. The 1-year and 2-years disease free survivals were 89.3% and 82.1% while overall survivals were 92.8% and 82.1% respectively.

**Conclusion:**

In developing countries like ours, relatively younger patients present to health center with obstructive colorectal cancer with anatomical shift to the right sided lesions. The treatments provided at our center and their outcomes are not inferior to that of the developed world.

## Introduction

Colorectal cancer can be considered a marker of socio-economic development, being common in developed world [1]. An increasing trend in the incidence of colorectal cancer (CRC) has been observed in Asia [2]. The increase in formerly low-risk and lower Human Development Index countries likely reflects changes in lifestyle factors and diet, i.e., shifts toward an increased intake of animal-source foods and a more sedentary lifestyle, leading to decreased physical activity and increased body weight. Additional risk factors include heavy alcohol consumption, cigarette smoking, and consumption of red or processed meat.

A word of caution must be spent with regards to the increasing incidence of CRC in the population younger than 50 years of age: this could potentially encourage an update in screening program [3]. To mitigate the rising burden of early-onset colorectal cancer, the American Cancer Society lowered the recommended age for screening initiation for individuals at average risk from 50 to 45 years in 2018 [4]. Most commonly, CRC patients present in the outpatient settings with changes in bowel habits, rectal bleeding and iron deficiency anaemia while in the emergency setting with the bowel obstruction. Partial or complete colonic obstruction occurs in 7% to 29% of patients with colorectal cancer [5]. Obstruction due to colorectal cancer represents challenging matters in terms of diagnosis, life-saving strategies, obstruction resolution and oncologic challenges. It could be fatal too because of perforation peritonitis or sepsis. Hence, the strategies for management of obstructive colorectal cancer must be based on patient characteristics, current cancer treatment and care goals.

CRC is the seventh most common cancer in Nepal, and the fifth among the cancer deaths [6]. The age-standardized incidence and mortality rates of CRC in Nepal were 5.8 and 4.8 per 100,000, respectively, in the year 2018 [6]. The health-seeking behaviors of CRC patients is very poor. A study in Western Nepal among 800 participants showed that 20% of them had not heard of CRC and only a few knew about CRC screening [7]. Screening of neoplasms such as cervical cancer and breast cancer are addressed in national public health programs. However, the CRC screening is not yet addressed effectively in the public health system of Nepal.

We opted to perform this study with aim to explore the treatment strategies being followed for patients with obstructing colorectal cancer (OCRC) at our institute and to know the management outcomes. The result of this study may be helpful to guide National Cancer Registry Program (NCRP) and help to know any change in the trend of CRC profile. This study will help the public health professionals, clinicians and policymakers on successful planning to address CRC.

## Materials and methods

Ours is a retrospective observational study, which included all patients diagnosed with Obstructive (partial or complete) colorectal cancer either pre-or intra-operatively over the 5-year period (January 2014 to December 2019) and managed at our academic institute.

Any patient presenting to us with distension of the abdomen, abdominal pain, nausea/vomiting and inability to pass stool ± flatus was considered to have bowel obstruction (**PARTIAL,** if passing flatus but no stool; **COMPLETE,** if not passing both stool and flatus).

All patients presented to us with bowel obstruction (either partial or complete) were advised abdominal X-ray series. If the patient had a complete bowel obstruction, abdominal X-ray showed significant air-fluid level and /or clinical examination revealed peritonism, he/she was considered to have an Emergency Surgical Condition which required urgent emergency laparotomy.

If the patient had a partial obstruction, he/she was considered to have a Semi-emergency Surgical Condition in which we had bought some times for additional radiological investigations (such as Ultrasound abdomen, CT-scan), colonoscopy ± biopsy, and Serum CEA on individual basis after admission in surgical ward. Then, they were posted for surgical intervention in the form of laparostomy.

The division into Emergency and Semi-emergency surgical conditions necessarily reduced the investigational burden in the emergency department in counties like ours with limited resources.

**Fig. 1:**
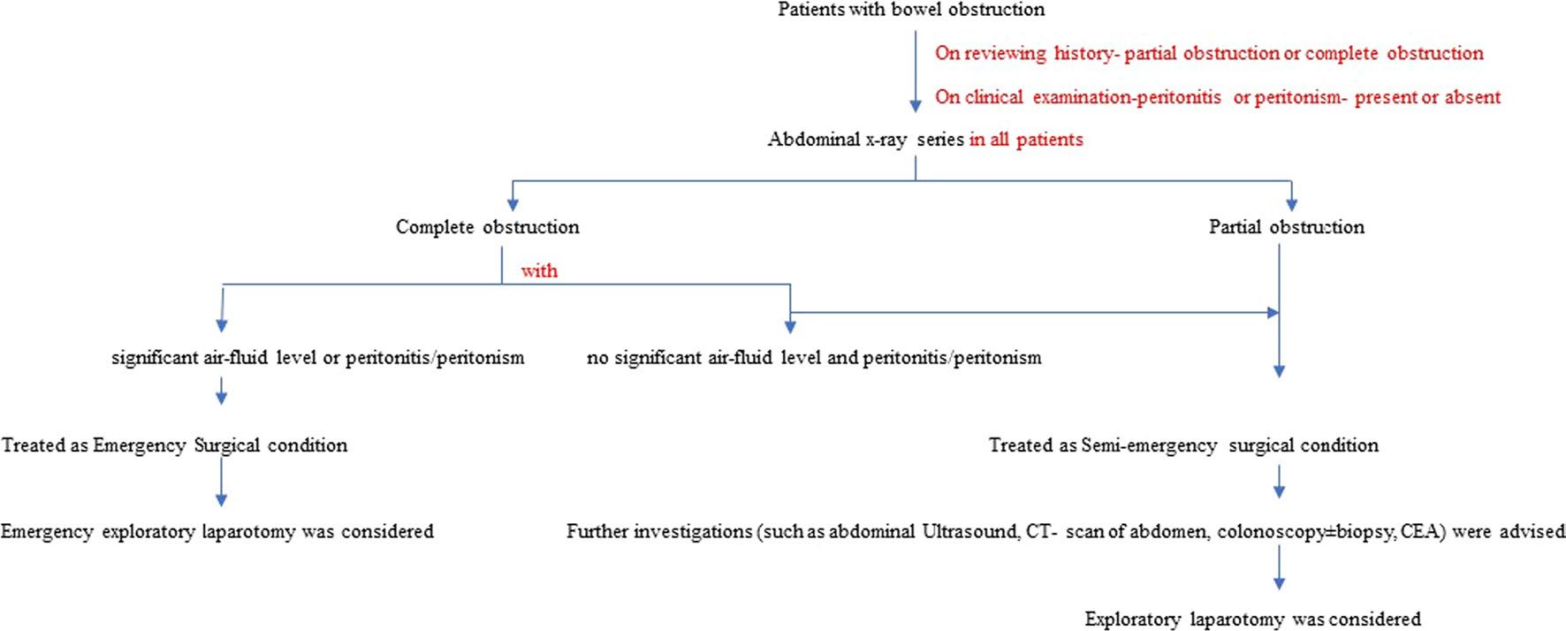
Scheme of treatment strategies of the bowel obstruction followed at our institute. *Emergency surgical condition* required urgent emergency exploratory laparotomy and proceed; *Semi-emergency surgical condition* provided some time for further investigations and laparotomy was performed thereafter

Approach that we followed to a patient with bowel obstruction presenting to our center has been described below in Fig. 1.

Only those patients in which the cause of obstruction were CRC (diagnosed either preoperatively with imaging studies or intraoperative incidentally) were included in study.

The patients’ medical records were reviewed to collect the following data: demographic and clinical information, blood investigations and radiological investigations performed, co-morbidity, pre-operative working diagnosis, operation performed and intra-operative finding.

The outcomes including post-operative complications, length of hospital stay and mortality were reviewed. The histopathological reports of resected specimen were reviewed to collect information about histological type, pathological stage, primary location of tumor, synchronous lesions, and resected margin status (Both proximal and distal margins). Any adjuvant therapy if given were noted. 2-years DFS and OS were calculated.

Data were entered in an excel sheet and converted into Statistical Package for Social Sciences software (SPSS version 17.0) for descriptive statistical analysis by calculating the mean, median (range) and percentage where appropriate.

### Sample size calculation

Total number of colorectal cancer cases managed at our institute over last 5 years period (2015–2020 AD) as per medical record section was 100; of which, 28 cases of CRC presented to us with bowel obstruction.

The study considered 95% Confidence Interval (CI) and 80% power to estimate the sample size. In literature, partial or complete bowel obstruction reported to occur in 7–29% of patients with colorectal cancer [5]. *Now taking average of this occurrence i.e., 18% and using one proportion formula for sample size calculation, we found that-*

$$n = \frac{{{\text{z}}2{\text{pq}}}}{{{\text{L}}2}}{\text{where z}} = 1.96\,{\text{at}}\,95\% \,{\text{CI}},{\text{p}} = 18,{\text{q}} = 82\,{\text{and}}\,{\text{L}} = 20\% \,{\text{of}}\;{\text{p}} = 3.6$$

$$= 445$$

Here, the sample size became 445, but it is a rare disease. So, using finite sample size estimation formula to estimate appropriate sample size, we found that

$${\text{n}} = \frac{{\text{calculated sample size}}}{{1 + \frac{{\text{calculated sample size}}}{{\text{estimated population}}}}}{ = 26}$$

where calculated sample size = 445 and estimated population = 28.

Adding 5% at calculated sample size to reduce various biases, it becomes around 27.

We had total of 28 cases that met the inclusion criteria of the study and hence all were enrolled in the study.

## Result

A total of 28 patients who were diagnosed with OCRC were included in the study. The demographic profile has been summarized in Table 1.

**Table 1:** Demographic profile of patients with OCRC

Characteristics	Frequency, n (%)
Age	
Mean age	49.79 ± 15.96 years
Age less than 40 years	6(21.4%)
Age more than and equal to 40 years	22(78.6%)
Gender distributions	
Male	14(50%)
Female	14(50%)
Location from where they belong to	
Urban area of eastern Nepal	12(42.9%)
Rural area of eastern Nepal	16(57.1%)

The clinicopathological profile of patients with OCRC has been summarized in Table 2.

**Table 2:** Clinicopathological profile of patients with OCRC

Parameters	Frequency, n (%)
Presenting features	
Abdominal pain Altered bowel habit Blood in stool Abdominal distension Anorexia Abdominal mass Weight loss Anemia Tenesmus Vomiting Bleeding per rectum	14(50%) 13(46.4%) 11(39.3%) 7(25%) 6(21.4%) 5(17.9%) 5(17.9%) 5(17.9%) 4(14.3%) 4(14.3%) 4(14.3%)
Obstruction	
Partial Complete	22(78.6%) 6(21.4%)
Commonly used relevant investigations	
Abdominal X-ray Fecal Occult Blood Test Ultrasound abdomen CT-scan abdomen Colonoscopy CEA (carcinoembryonic antigen) Pre-operative biopsy Post-operative biopsy	28(100%) 1(3.6%) 26(92.8%) 24(85.7%) 22(78.6%) 21(75%) 8(28.6%) 28(100%)
Primary location of tumor	
Rectosigmoid Descending colon Splenic flexure Hepatic flexure Ascending colon Caecum	10(35.2%) 2(7.2%) 1(3.6%) 6(21.6%) 5(18%) 4(14.4%)
Synchronous lesion	
Yes No	8(28.6%) 20(71.4%)
Histological types	
Adenocarcinoma	28(100%)
Pathological staging	
Stage 1 Stage 2 Stage 3 Stage 4	3(10.7%) 1(3.6%) 15(53.6%) 9(32.1%)
Tumor-free Margins of Resected specimen	
Yes No	28(100%) 0(0%)
Number of lymph nodes harvested in specimen	
< 12 > / = 12	8(29%) 20(71%)

The treatments provided at our center and their outcomes have been summarized in Table 3.

**Table 3:** Treatments provided to patients with OCRC and their outcomes at our center

Characteristics	Frequency, n 1(%)
Operation performed	
Right hemicolectomy Extended right hemicolectomy Left hemicolectomy Extended left hemicolectomy Anterior resection Abdominoperineal resection Hartmann’s procedure	9(31.6%) 6(21.6%) 2(7.2%) 1(3.6%) 3(10.8%) 5(18%) 2(7.2%)
Post-operative complications	
Surgical site infection Hospital acquired pneumonia Anastomotic leak Paralytic ileus Diarrhea	7(25.2%) 4(14.4%) 2(7.2%) 2(7.2%) 1(3.6%)
Post-operative mortality	
No Yes	27(96.4%) 1(3.6%)
Hospital stay (mean ± SD)	13.82 ± 6.87 days
Adjuvant chemotherapy (FOLFOX)	
Yes No	18(64.3%) 10(35.7%)
Disease free survival	
1 year 2 years	25(89.3%) 23(82.1%)
Overall survival	
1 year 2 years	26(92.8%) 23(82.1%)

## Discussion

Though the incidence of obstructive colorectal cancer (OBCR) previously reported to peak in the seventh and eighth decades of life [8], it is nowadays increasing in the younger age group especially in Asian countries, with mean age of 49.78 ± 15.96 years in our study with 1/4th of the patients being younger than 40 years of age. However, there continues to have no difference in the incidence of OCRC among genders that is, 14(50%) males and 14(50%) females in our study.

The incidence of CRC has been higher in the urban areas of economically advantaged countries [9]. This is thought to be related to consumption of a high fat/ high red meat and obesity. In country like ours, scenario is different, being common in rural areas. There can be two separate reasons for this occurrence: one, lack of awareness of CRC & its screening program implementation; and two, delay presentation of patient to the health care system in the rural areas.

Clinical features of colorectal cancer may vary significantly depending on the anatomical site and disease stage of the tumor. Most common presenting feature in our patients was abdominal pain in 14(50%) patients followed by altered bowel habit, blood in stool, abdominal distention, anorexia, abdominal mass, weight loss, anemia, tenesmus, vomiting and bleeding per rectum respectively, similar to other studies in literature [10]. Overall, of 28 patients with OCRC, 21.4% patients had complete bowel obstruction while remaining had partial bowel obstruction. However, atypical presentations such as pyogenic liver abscess, intussusception, discitis have also been reported [11, 12]. Various diagnosing modalities used in our study included abdominal X-ray (in 100% cases), ultrasonoghraphy of abdomen and pelvis (in 92.8% cases), abdominal CT-scan (in 85.7% cases), colonoscopy (in 78% cases), serum CEA level (in 75%cases), pre-operative punch biopsy (in 28.6% cases) and Faecal Occult Blood test (in 3.6% cases). These all-investigating modalities need to be individualized as per the way patients present to us and the availability of resources specially in context to the developing countries like ours.

Theoretically, distal colonic cancers are more prone to present with the bowel obstruction because proliferative type of lesions are common in such location and the stool are of semisolid consistency. Meanwhile the proximal colon cancers are usually ulcerative and the stool in this area will be of relatively liquid consistency. However, the incidence of proximal CRC causing obstruction has been increasing nowadays [13]. In our study, in 54% cases the lesion was in the proximal colon. This shift may be because of genetic factors, which can preferentially involve defects in mismatch repair genes with resulting microsatellite instability in proximal colon cancers whereas chromosomal instability pathway is common in left sided colorectal cancers. This anatomical shift will necessarily impact on screening policy [14].

The overall incidence of synchronous lesions in CRC has been reported to be between 2.3% to 12.4% in literature [15]. However, its exact incidence in CRC patients when they develop bowel obstruction has not yet been mentioned. In our study, a higher incidence of synchronous lesions up to 28.6% has been found in patient with CRC presenting to us with the bowel obstruction. The likely explanation can be that once patients with CRC develop obstruction, often they have advanced stage disease. It has been claimed that apart from obstructive symptoms, other symptoms do not necessarily correlate with stage of disease [16].

In our study, the most common histological type was adenocarcinoma 28 (100%). The extent of resection should be based on the primary location of cancers and the principle oncological resection: no touch technique, high ligation of vessels and total mescolon/mesorectal excision should be followed to get a standard quality specimen with adequate number of lymph nodes harvested in the specimen. In our study, $\ge$ 12 lymph nodes were harvested in 20 out of 28 specimens.

Bowel obstruction in patients with CRC occurs when a cancerous growth or adhesion block intestinal flow. Most of patients in our study had advanced stage disease: stage 3 in 53.6% cases followed by stage 4 in 32.1% cases. Interestingly, 10% patients with OCRC were found to have stage 1 disease. The possible explanation of bowel obstruction in stage 1 disease, can be a physiological inability to move the food particles in addition to general concept of cancerous growth obstructing the lumen of bowel.

The various treatment options for OCRC opted at our center were right hemicolectomy, extended right hemicolectomy, left hemicolectomy, extended left hemicolectomy, anterior resection, abdominoperineal resection and Hartmann’s procedure. The common post-operative complications encountered at our study were surgical site infection, hospital acquired pneumonia, anastomotic leak, prolong paralytic ileus and diarrhea. We lost one patient in post-operative period and it accounts to post-operative mortality rate of 3.6% in our study which very less in comparison to the study by Kaya et al. [17]. Patients averaged 13.82 days in the hospital (SD 6.87 days) which is also almost half of what had been found in study by Kaya et al. [17]. Not all but 64.3% patients with OCRC received adjuvant chemotherapy at our center. The 1-year and 2-years disease free survivals were 89.3% and 82.1% while overall survivals were 92.8% and 82.1% respectively. These are comparable with data from more developed nations [18].

## Conclusion

In developing countries like ours, many patients with colorectal cancer continue to present to health center in advanced stage with bowel obstruction. CRC, nowadays, is not ***uncommon*** in patients younger than 40 years of age in our country and the incidence of the proximal lesion causing obstruction is increasing***.*** The treatments provided at our center and their outcomes are not inferior to that of the developed world.

## Recommendation

A word of caution must be paid with regards to implementation of National Colorectal Cancer Screening program strongly and at relatively early age of life.

## Limitation

The few limitations of this study are retrospective type, small sample size and single centered study.

## Data Availability

The datasets used and/or analysed during the current study available from the corresponding author on reasonable request.
